# Impact of immune-mediated diseases in inflammatory bowel disease and implications in therapeutic approach

**DOI:** 10.1038/s41598-020-67710-2

**Published:** 2020-07-01

**Authors:** M. J. García, M. Pascual, C. Del Pozo, A. Díaz-González, B. Castro, L. Rasines, J. Crespo, M. Rivero

**Affiliations:** Gastroenterology Department, Marques de Valdecilla University Hospital - IDIVAL, Santander, Cantabria Spain

**Keywords:** Epidemiology, Applied immunology, Autoimmunity

## Abstract

Inflammatory bowel diseases (IBD) belong to the group of immune-mediated diseases (IMIDs). The effect of associated IMIDs in the prognosis in IBD is nowadays unknown. To describe IMIDs associated to IBD patients and evaluate differences linked to the presence or absence of IMIDs. A unicentric retrospective descriptive study was designed. A cohort of 1,448 patients were categorized according to the presence of IMIDs. Clinical characteristics were obtained from IBD database. Univariate and multivariate analysis were performed. 385 patients were diagnosed with associated IMIDs while 1,063 had no associated IMIDs. A prevalence of 26.6% IMIDs associated to IBD was observed. Asthma, skin psoriasis and rheumatoid diseases were most commonly found. Factors associated to the presence of IMIDs were women (OR 1.48; 95 CI 1.17–1.87) and Crohn’s disease (OR 1.35; 95 CI 1.07–1.70). Patients with associated IMIDs required more immunomodulator (OR 1.61; 95 CI 1.27–2.43) and biological treatment (OR 1.81; 95 CI 1.47–2.43). More surgical risk was observed in multivariate analysis in those patients diagnosed with IMIDs prior to the onset of IBD (OR 3.71; 95% CI 2.1–6.56). We considered the presence of IMIDs a poor prognostic factor and suggest a closer monitoring of these patients.

## Introduction

Immune mediated diseases (IMIDs) are a group of autoimmune inflammatory disorders characterized by an alteration in cellular homeostasis^1^. The etiology of these diseases is multifactorial including environmental factors, dietary habits and infectious agents in patients with a genetic predisposition^2^. This causes tissular injury of the organ affected due to the excessive inflammatory reaction^3,4^. In that way, the loss of tolerance of the system to autoantigens and environment antigens originate the uncontrollable production of antibodies or the denominated “phenomenon of autoreactivity”^5^.

To avoid this situation, there are immunoregulatory cytokines that try to control the cellular homeostasis and control the balance of the system; CD4 + T lymphocytes belong to this system. Depending on the antigen presented by antigen-presenting cells (APC), lymphocytes become TH1 or TH2 response. Overall, TH1 response has a pro-inflammatory effect in autoimmune disorders and increase the production of pro-inflammatory cytokines like TNF-α^6^. In IMIDs, there is a TH1 main reaction and also a reduction of the differentiation to TH2 response on the part of T lymphocytes^7^. Once an antigen in IMIDs initiates the inflammatory response, a higher production of pro-inflammatory cytokines is observed as TNF-α, IL-1, IL-6 and the reduction of other cytokines such as IL-10 and IL-4^8,9^.

Regarding the group of IMIDs, all the diseases with autoimmune origin and essentially TH1 response are included. Among these diseases, different specialities as Endocrinology, Gastroenterology, Hepatology, Rheumatology and Dermatology are involved provided that the complexity of these pathologies usually demanding a multidisciplinar team for the evaluation and monitoring of these diseases. Inflammatory bowel diseases including Crohn’s disease, ulcerative colitis and IBD-unclassified, are some of the immune-mediated diseases which affect up to 2.2 million patients in Europe with an annual incidence ranging between 9 and 15% depending on the geographical area^10–12^. Recently, an annual incidence of 9%-20% of IMIDs associated to IBD was described as well as its association with extraintestinal manifestations and family history of IBD^13^. However, there is a lack of knowledge related to the association between IMIDs and IBD and their influence in the evolutive course of these diseases and also the therapeutic requirements of immumomodulators or biological therapy^14^. Most of the studies described the association between IBD and thyroid disorders^15–17^, skin psoriasis among others diseases and different conclusions are discussed about prognostic implications of these diseases in IBD^18,19^. Furthermore, recent data showed the need of more surgical procedures in patients with IMIDs even other studies showed disparities with the results^20^. For this reason, we designed a study with two essential aims: on the one hand, to describe IMIDs associated to IBD in a cohort of patients and on the other hand, the clinical differences and therapeutic requirements in relation to the presence or lack of IMIDs in said patients.

## Methods

A retrospective cohort study was carried out. Patients were collected in an IBD local database with retrospective data collection until 2015 and followed by a prospective data collection up until today. The local database used for IBD patients treated in our tertiary referral hospital is updated regularly. The medical history of all patients was also updated at the time in which the data was collected. The informed consent was obtained from the patients for including clinical characteristics of IBD. At the time of the study, an exhaustive evaluation of the associated IMIDs were reviewed in past medical history entries from different Specialists and General Practitioners (GP). IMIDs data collection was gathered from 20th June of 2018 to 31st October of 2018. The study was carried out in accordance to the General Data Protection Regulation 2016/679 (GDPR) and it was approved by the Ethical Committee of Cantabria.

All patients were diagnosed with IBD according to 3 categories organized in Crohn’s disease, ulcerative colitis and IBD-unclassified according to the recommendation set by the European Crohn and Colitis Organization (ECCO)^21,22^. Demographic characteristics were recorded such as IBD family history, smoking habit at the time of the study, associated complications and clinical characteristics as well as the diagnostic date of both IMIDs and IBD. Patients were considered current smokers when they smoked more than 7 cigarettes per week, former smokers when they last smoked 6 months before and non-smokers when the patient had never smoked before^23^.

The location and behaviour of the diseases were classified according to the Montreal Classification^24^. With regards to drug administration, we studied the indication of the treatment, immunosuppression, biological therapy and surgeries needed during the course of the disease. IMIDs were defined according to World Health Organization International Classification, 2018 edition^25^. We excluded from the analysis any paradoxical effect of the drugs such as psoriasis, lupus or secondary arthritis due to anti-TNF treatment.

Firstly, a descriptive analysis was performed in all patients diagnosed with IBD. To establish the comparative groups, they were classified in two: IBD patients with and without associated IMIDs. Both groups were classified according to age in 3 groups: less than 40 years old, between 40 and 60 and more than 60. Once the patients were grouped, the clinical characteristics of both categories were studied and differences concerning clinical features, demographic records and disease evolution were examined. IBD complications were defined as the presence of abdominal abscess, gastrointestinal bleeding which required blood transfusion, perforation or megacolon. Afterwards, IBD diagnosis before or after the diagnosis of IMIDs were also compared to analyse their influence in the progression of IBD.

A statistical analysis was performed with mean and standard deviation for continuous variables and percentages for qualitative variables. Chi test was used by qualitative variables while quantitative variables between two groups were analysed by Student T-test. A multivariate analysis through logistic regression to calculate odds ratio (OR) was made to compare the risk of every variable with respect to the reference group choosing a confidence interval of 95% and an alpha error of 5%. The analysis was performed separately for each variable and afterwards, a multivariate analysis by backward regression was done to evaluate confounder factors. Statistic significant variables and those that were clinically relevant were evaluated in the multivariate analysis. A significant result was considered when the “p” was < 0.05. The cumulative incidence of surgery after IBD diagnosis was estimated using Cox regression. All statistical analyses were performed with STATA Statistical Software: Release 14. StataCorp LP.

## Results

A retrospective cohort was conformed in our group of patients. Our sample was established by 1,448 patients, of which 26.6% (n = 385) had an associated IMID meanwhile 73.4% (n = 1,063) did not have associated IMIDs. A percentage of 50.8% (n = 735) were men meanwhile 49.2% (n = 713) were women. A prevalence of 30.7% of IMIDs was recorded in the group of women with regards to 22.9% in the group of men with a statistic significance of 0.001. The mean age of the patients of our cohort at the time of the study was 53.9 years (SD 15.45). Attention has to be drawn to the fact that the mean IMIDs diagnostic age was previous to the mean IBD diagnosis age (39.2 years (SD 15.85) versus 40.7 years (SD 16.12) (p = 0.04)). No statistical differences in the IMIDs proportion was observed according to the three groups of age above mentioned. Indeed, there were no differences in the mean age when IBD started in both groups. There was a proportion of 46.9% (n = 680) affected by Crohn’s disease, 48.3% (n = 700) by ulcerative colitis and 4.7% (n = 68) of patients with IBD-unclassified. Clinical and demographic characteristics are detailed in Table 1.

**Table 1:** Demographic and clinical characteristics of the patients.

	Total (n = 1,448)	Ulcerative colitis (n = 700)	Crohn disease (n = 680)
Gender: men (n, %)	735 (50.8)	344 (49.1)	352 (51.8)
Age (years) (mean, SD)	53.83 (15.4)	55 (15.1)	52.68 (15.6)
Diagnostic age of IBD (years) (mean, SD)	40.70 (16.1)	42.42 (15.2)	38.50 (16.6)
Average duration of IBD (years) (mean, SD)	13.13 (9.7)	12.58 (9.3)	14.17 (10.2)
Average duration of IMIDs (years) (mean)	15.6 (4.3)	15.49 (4.2)	15.75 (4.5)
**Tobacco (n, %)**			
Current smokers	233 (18.4)	55 (9.1)	170 (28.2)
Former smokers	193 (15.2)	102 (16.9)	82 (13.6)
Non smokers	841 (66.4)	447 (74.0)	351 (58.2)
Familiar history (n, %)	92 (6.4)	34 (4.9)	55 (8.1)
Complications (intra-abdominal abscess, megacolon, bleeding, perforation) (n, %)	95 (6.7)	7 (1.0)	88 (13.4)
**Location (n, %):**			
Ulcerative proctitis		229 (32.7)	
Left-side colitis		296 (42.3)	
Extensive colitis		175 (25)	
Ileum (CD)			337 (49.6)
Colon (CD)			99 (14.6)
Ileocolonic (CD)			244 (35.9)
Upper disease (CD)			22 (3.2)
**Behaviour (n, %)**			
Inflammatory			445 (65.4)
Stricturing			108 (15.9)
Penetrating			127 (18.7)
Perianal disease (n, %)	153 (10.6)	24 (3.4)	122 (18.0)
**Therapy**			
Immunosupresor (n, %)	577 (40.0)	170 (24.3)	296 (58.2)
Biological therapy (n, %)	387 (6.7)	105 (15)	271 (39.9)
Surgery (n, %)	289 (19.9)	37 (5.3)	250 (36.8)

### Descriptive study: prevalence of IMIDs in patients affected by IBD

**Figure 1:**
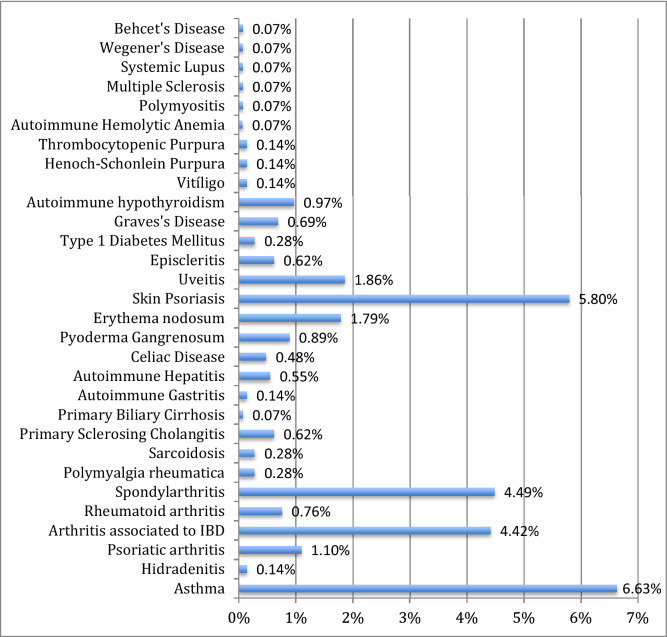
Prevalence of IMIDs associated to IBD.

In our cohort, a 26.6% (n = 385) prevalence of IMIDs was collected. The IMIDs most frequently described were rheumatologic disorders. In the cases where there was an associated IMIDs, one was described in 17.8% (n = 257) of the patients, two were described in 6.3% (n = 91), three in 2.21% (n = 32) and more than four in 0.4% (n = 5) of the patients. Intrinsic asthma was the most common IMID in patients with IBD in 6.6% (n = 96) followed by skin psoriasis in 5.8% (n = 84) and spondylarthritis in 4.5% (n = 65). Different proportions of IMIDs in patients with IBD are detailed in Fig. 1.

### Factors associated with IMIDs

In the group of associated IMIDs, there was a larger proportion of affected women measured by an OR of 1.48 (95% CI 1.08–1.87) in a statistically significant way. Also, it must be highlighted that IMIDs were frequently associated with Crohn’s disease compared to patients affected by ulcerative colitis (OR de 1.33; 95% CI 1.05–1.68). The duration of IBD was not correlated with the presence of associated IMIDs (*p* = 0.4). No differences between familiar history or tobacco habit were found in both diseases.

It should be noted that the use of immunomodulators was most frequent in IBD patients with associated IMIDs compared to isolated IBD (48.3% vs. 36.8%, *p* < 0.0001). This data is similar to the one obtained in patients treated with biological therapy with IMIDs associated to IBD who presented more use of these drugs compared to patients without IMIDs (36.3% vs. 23.2%, *p* < 0.0001). Therefore, patients with at least one associated IMID to IBD had a risk of 1.61 (95% CI 1.27–2.03) for receiving immunomodulator therapy and a risk of 1.89 (95% CI 1.47–2.43) for receiving biological therapy making this result statistically significant. We observed that immunomodulators were required in 45.1% of patients with only one IMID, 50.6% with 2 IMIDs and 64.9% for those patients with more than 3 IMIDs. On the other hand, biological therapy was necessary in 33.1% of the cases with one IMID, 38.5% with 2 IMIDs, and 54.0% for those with more than 3 IMIDs. Regarding the indication, immunomodulator prescription was initiated to control the inflammation in IBD in 95.2% of the patients. Furthermore, only 8.0% (n = 31) patients, who had received biological therapy, was due to some other associated diseases and not exclusively because of IBD. However, patients affected by concomitant peripheral arthritis received biological therapy in 51.6% and those with concomitant spondylarthritis in 52.3% (*p* = 0.001).

According to prognostic factors, there were no significant differences in surgery needs related to the coexistence of IMIDs (OR 1.10; 95% CI 0.82–1.46). In those patients affected by erythema nodosum, an OR of 3.02 (95% CI 1.24–7.11) of needing surgery was observed. No statistical significance was obtained in other diseases regarding these prognostic factors. Risk factors and their relationship with IMIDs are described in Table 2.

**Table 2:** Clinical and therapeutic features related to the presence of IMIDs.

	IBD with IMIDs associated	IBD without IMIDs	*p* value
	%, n	%, n	
**Gender**			
Men	43.6 (168)	53.3 (567)	< 0.01*
Women	56.4 (217)	46.7 (496)	
**Familiar history**			
Yes	6.5 (24)	6.4 (68)	0.91
No	93.5 (345)	93.6 (1,000)	
**Tobacco**			
Current	20.3 (69)	17.7 (164)	0.57
Former	14.7 (50)	15.4 (143)	
Non smoker	65 (221)	66.9 (620)	
**Type of disease**			
Crohn’s disease	52.2 (201)	45.1 (478)	0.02*
Ulcerative colitis	44.7 (172)	49.7 (528)	
**Complications**			
Yes	7.8 (29)	6.4 (66)	0.33
No	92.2 (342)	93.7 (973)	
**Immunomodulator therapy**			
Yes	48.3 (186)	36.8 (391)	< 0.01*
No	51.7 (199)	63.2 (672)	
**Biological therapy**			
Yes	36.4 (140)	23.2 (247)	< 0.01*
No	63.6 (245)	76.8 (816)	
**Surgery**			
Yes	21.0 (81)	19.6 (208)	0.75
No	79.0 (304)	80.4 (855)	

### Factors associated to IMIDs: differences between Crohn’s disease and ulcerative colitis

When we analysed the factors associated to the presence of IMIDs depending on the type of IBD, we found more risk for suffering associated IMIDs for Crohn’s disease as above mentioned. No specific pattern was associated with a higher risk of having IMIDs in Crohn’s disease even though there was an OR of 1.48 (95% CI 1.01–2.17) in patients with ulcerative colitis with pancolitis involvement. With regards to the treatment, we observed a statistical association between the coexistence of an IMID in ulcerative colitis and the prescription of immunomodulators (OR 1.82; 95% CI 1.25–3.34) and biological therapy (OR 2.16; 95% CI 1.4–3.34). This association was also described when biological therapy was administrated in patients affected by Crohn’s disease (OR 1.59; 95% CI 1.14–2.22). Differences between both diseases are described in Table 3.

**Table 3:** Factors associated to the presence of IMIDs: differences between Crohn’s disease and ulcerative colitis.

	UC with IMID associated (%, n)	Odds ratio (95% CI)	CD with IMID associated (%, n)	Odds ratio (95% CI)
Gender (Women)	61.1 (105)	1.73 (1.22–2.45)	52.2 (105)	1.26 (0.9–1.75)
Familiar history (yes)	3.5 (6)	0.64 (0.27–1.55)	9.0 (18)	1.17 (0.66–2.1)
Tobacco (smoker vs. no smoker)	10.7 (16)	1.19 (0.65–2.19)	28.9 (52)	1.19 (0.83–1.69)
Complications (yes)	1.2 (2)	1.22 (0–5.5)	14.2 (27)	1.1 (0.68–1.8)
Immunomodulator therapy (yes)	33.1 (57)	1.82 (1.25–2.66)	62.2 (125)	1.26 (0.9–1.77)
Biological therapy (yes)	23.3 (40)	2.16 (1.4–3.34)	47.8 (96)	1.59 (1.14–2.22)
Surgery (yes)	7.0 (12)	1.5 (0.75–3.04)	34.3 (69)	0.86 (0.61–1.21)

### The onset of an IMID previous to IBD is a poor prognostic factor for surgery requirement in patients with IBD

In our study, we also evaluated the presence of IMIDs and IBD attending to the time of diagnosis. IMIDs were diagnosed in 15.8% (n = 228) of the patients prior to the diagnosis of IBD. The mean diagnostic age of IBD was 54.04 years (SD 15.86) when an IMID was previously diagnosed to IBD meanwhile in the group with IBD prior to IMID, the mean diagnostic age of IBD was 57.08 years (SD 12.81). We did not identify statistically significant differences regarding the time interval between IMID and IBD diagnosis when comparing both groups (9.2 years (SD 11.8) for IMIDs previous to IBD and 8.2 years (SD 7.53) for IBD previous to IMIDs) (*p* = 0.38).

**Figure 2:**
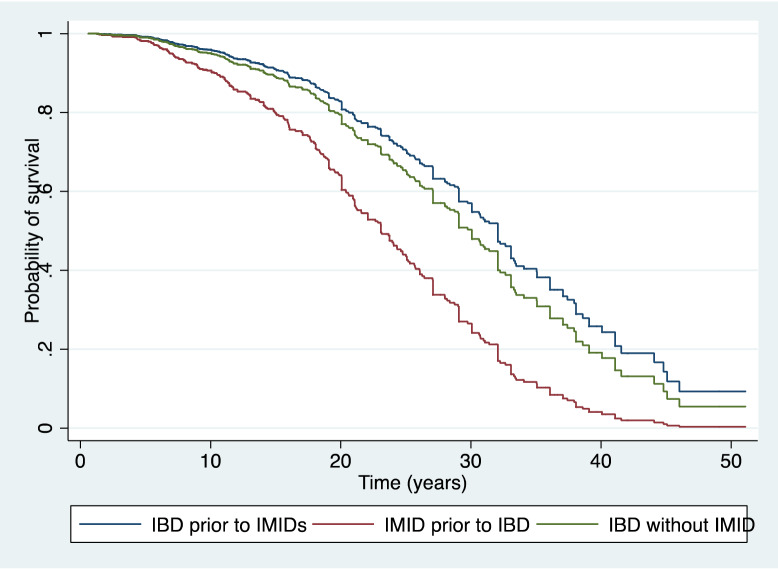
Cox proportional hazard survival curves according to IMIDs diagnosis prior to IBD, IBD diagnosis prior to IMIDs or patients without IMIDs associated.

We identified a prognostic profile attending to the moment of diagnosis. Those patients diagnosed of an IMID previous to IBD presented an aggressive IBD pattern, measured by an earlier surgical approach (HR 3.02; 95% CI 1.75–5.23) (Fig. 2). Furthermore, we identified a higher risk of surgery in patients under biological therapy, perianal disease and complications in the univariate analysis (Table 4). Finally, the multivariate analysis showed that being diagnosed of a previous IMID to IBD predicts worse outcomes, as these patients are in need of an early surgical approach with an HR of 3.71 (95% CI 2.10–6.56) (Table 4). The most frequently associated IMIDs before the diagnosis of IBD were asthma in 34.7% of patients, skin psoriasis in 21.9%, arthritis in 14.5%, spondylarthritis in 14.9%, uveitis in 6.6% of the patients and erythema nodosum in 6.1% of the patients.

**Table 4:** Risk factors for surgery in patient with IMIDs in univariate and multivariate analysis.

	Univariate analysis		Multivariate analysis	
	Adjusted hazard ratio	CI	Adjusted hazard ratio	CI
IMIDs prior to IBD	2.36	1.45–3.85	3.71	2.1–6.56
Complications	2.44	1.47–4.06	1.07	0.56–2.07
Perianal disease	1.63	1.00–2.68	1.7	1.03–2.79
Immunomodulator therapy	1.19	0.92–1.55	0.47	0.25–0.88
Biological therapy	0.85	0.65–1.23	2.17	1.24–3.82

## Discussion

An observational study of cohorts was performed to describe the influence of IMIDs in IBD, their implication in the progression of the disease and available treatments. In our study, we observed a prevalence of IMIDs of 26.6%, higher than the prevalence described in previous studies conducted in different geographical areas^26–28^. However, an increased incidence of these diseases has been described in IBD patients currently reaching similar prevalences compared to our findings^29^. Only a few studies have evaluated IMIDs in patients with IBD. In our cohort, asthma was the most prevalent disease followed by skin psoriasis and spondylarthritis. These results are similar to data that was recently described in other studies^30,31^. Lolli et al. identified a frequency of skin psoriasis at less than 25% and plaque psoriasis was the most commonly described^32^. Regarding spondylarthritis, a prevalence of more than 10% was also identified in patients with IBD, a result consistent with the data obtained in our study^33^.

It is well known that a worst quality of life exists in IBD patients with associated IMIDs as a result of disease activity, comorbidity and drug side effects^34^. Considering this limitation in the quality of life for the patients, it is essential to recognize prognostic factors and the influence of IMIDs in the disease evolution of IBD. Albeit there are variety of data, it seems that the risk of IMIDs is higher in patients affected by both diseases with regards to the general population^35^. The highest risk of having IMIDs was recorded for Crohn’s disease with an incidence ratio of 147 every 100,000 inhabitants per year^36^. No relationship was found between the behaviour or location of the disease^37,38^. Nevertheless, in patients with ulcerative colitis, an increased risk of IMIDs was observed in those patients with extensive colonic involvement according to similar data published by Conway^20^. While tobacco habit is a factor of poor prognostic in Crohn’s disease and other immune-mediated diseases such as skin psoriasis, no association was observed in our cohort^39–41^. Few studies have suggested that the impact of IMIDs on IBD prognosis varies based on the type of IMID^42^. However, we only found a higher risk of surgery in patients affected by erythema nodosum.

Immunosupresive treatments are a group of drugs commonly used in diseases with autoimmune characteristics like IMIDs^43^. Therefore, there are specific drugs that are used in many IMIDs due to their shared pathophysiological mechanisms. That is the case of methotrexate, azathioprine, anti-TNF and IL-12, IL-23 and IL-17 inhibitors^44–47^. In our cohort, suffering both diseases were associated to an increased use of immunomodulator and biological therapy. That risk was appreciated as well when they had concomitant peripheral arthritis and skin manifestations such as psoriasis, pyoderma gangrenosum and erythema nodosum. The use of biological therapy might be facilitated in the previously mentioned cases due to the effectiveness of the treatments to control several diseases. However, the clinical response to these treatments were worst in patients affected by associated IMIDs than those with isolated IBD, measured by the number of treatments needed to control the disease. Similar data regarding the biological treatment in patients with associated IMIDs was published^20^. Despite the facts, we should consider that sometimes, paradoxical reactions to these drugs appear^48^. In our study, psoriasis or arthritis secondary to biological treatments were specifically excluded. Nowadays, very few studies evaluate whether the early use of biological treatments could modify the incidence of IMIDs associated to IBD and therefore, a better evolution of the disease could be obtained.

It is important to highlight that IMIDs were previously diagnosed to IBD in a percentage of 15.8% in our study. Furthermore, our patients were classified according to behaviour and location of the disease enabling us to find a correlation between the subsequent IMIDs and IBD behaviour. Although smoking habit, early age, perianal disease and penetrating behaviour are poor prognostic factors for surgery in IBD, only perianal disease was associated to higher risk of surgery in those patients with previous IMIDs to IBD in the univariate analysis^49^. Only a recent study published by Burisch et al. described data about the temporality of IBD-IMIDs in the scientific literature finding a worse clinical evolution of IBD in those patients with at least 1 concurrent IMID to IBD^50^. Therefore, a patient affected by an IMIDs could require a closer monitoring of inflammation when IBD appears, especially if there is a Crohn’s disease. Biological therapy is commonly used for refractory IBD. For this reason, it is no strange that patients under biological therapy require more surgery to control the disease. Additionally, we found that the diagnosis of IMID and the following onset of an IBD was statistically related to experience a more aggressive evolution of the disease measured by the needs of surgical procedures in the multivariate analysis. This data could be justified as the presence of an IMID before the diagnosis of IBD could indicate that there is an excess of TH1 activation pathway and the absence of inflammation control brought by the drugs prescribed for IMIDs^51^. This is probably a consequence of the huge inflammatory charge that IBD has compared to other inflammatory diseases and the different autoinflammatory pathways implicated in their pathogenesis^52^. Despite that the presence of perianal disease and the use of biological therapy were associated with aggressive forms of the disease, the temporality of IMID was the most important factor that justified the need for surgery. However, this fact could be affected by the age because the beginning of IBD before 40 is generally related to suffering an aggressive course of the disease^53^. It should also be taken into consideration that in our sample, the patients with IBD previous to IMIDs were diagnosed with IBD more than 6 years before than the IMIDs previous to IBD group. It is not yet known if patients diagnosed by IMIDs will develop IBD in the future because of the retrospective nature of our data collection. Therefore, futures studies comparing both samples will be necessary.

Our study has some limitations. These include the retrospective method used for data collection with the consequent restrictions like the absence of information of corticosteroids prescription. Additionally, the use of biological therapy could be higher than in other centres as our hospital is a third level one and the referral for patients with more aggressive diseases. One of the greatest challenges in our study was the selection of diseases to be included in IMIDs. Nowadays, there is no common agreement in the types of diseases included in the IMID category and the studies published are not homogeneous regarding this definition. For our study, any disease with autoimmune and inflammatory involvement, which could have a systemic effect in the subject was considered an IMID. Regarding extraintestinal manifestations, extensive data was published where it was shown that these manifestations were associated with a poor prognostic of the IBD^54^. We considered that extraintestinal manifestations belong to the IMID group because IMIDs represent a widest group of diseases associated to the TH1 inflammatory pathway and are frequently overlapped with extraintestinal manifestations.

In conclusion, our study shows a prevalence of IMIDs associated to IBD of more than 20%. It is essential to recognize associated prognostic factors and the influence that IMIDs have in the evolution of the patients with IBD. Furthermore, those patients diagnosed with IMIDs associated to IBD will require a higher use of immunomodulators and biological therapy. The diagnostic of previous IMIDs to IBD was associated with a higher risk of surgery. Therefore, we should consider the presence of IMIDs associated to IBD as a poor prognostic factor and suggest a closer monitoring of these patients.

## Abbreviations

IMIDs: Immune-mediated diseases
IBD: Inflammatory bowel disease
ECCO: European Crohn’s ad Colitis Organisation
APC: Antigen presenting cells
TH1: T helper lymphocytes type 1
TH2: T helper lymphocytes type 2
TNF-α: Tumor necrosis factor alpha
IL: Interleukin
GP: General practitioners
SD: Standard deviation
OR: Odds ratio
